# Impact of Emergency Department Intravenous-Fluid Conservation Strategies During a National Shortage: Multisite Retrospective Study

**DOI:** 10.5811/westjem.49038

**Published:** 2026-01-26

**Authors:** Hannah Moreira, Ross McCormack, Cecilia Sorensen, Brandon Mallory

**Affiliations:** * New York Presbyterian/Columbia University Irving Medical Center, Department of Emergency Medicine, New York, New York; † Columbia University, Mailman School of Public Health, Department of Environmental Health Sciences, New York, New York

## Abstract

**Introduction:**

Effective disaster response in healthcare depends on coordinated strategies that maintain access to critical supplies across institutions. During Hurricane Helene in September 2024, a major intravenous (IV) fluid shortage caused by the destruction of a manufacturing plant exposed the vulnerability of centralized supply chains. Our objective in this study was to evaluate the impact of a multisite IV fluid conservation initiative on ordering patterns, cost, and environmental outcomes across three emergency departments (ED).

**Methods:**

We conducted a retrospective study evaluating large-volume, IV fluid-bolus orders placed before, during, and after the critical shortage. Interventions included an interruptive alert in the electronic health record, clinician education, and workflow adjustments. Our primary outcome measure was the number of IV fluid-bolus orders placed during each period. Secondary outcomes included total fluid volume administered, total cost of fluids, estimated carbon dioxide emissions, and the proportion of ED encounters involving fluid administration.

**Results:**

During the pre-shortage period, 24,251 IV fluid-bolus orders were placed across 41,752 ED encounters (41.8%). Orders dropped to 18,692 during the critical shortage across 39,840 encounters (30.8%), reflecting a 22.9% relative reduction. In the post-shortage period, 23,911 orders were placed across 40,967 encounters (39.6%), remaining slightly below baseline. Estimated cost savings during the shortage period totaled $27,202, with a projected annual savings of $108,808. Carbon dioxide emissions dropped by 3.1 metric tons—the equivalent of avoiding the use of over 349 gallons of gasoline.

**Conclusion:**

Emergency department-based conservation strategies were associated with measurable reductions in IV fluid use, cost, and environmental impact. Further validation is needed to understand their impact on clinical outcomes and healthcare system resilience.

## INTRODUCTION

The stability of the United States’ healthcare supply chain is essential for effective emergency medical response, yet it faces mounting pressure from escalating climate-related disasters. Extreme weather events are becoming more frequent and severe due to climate change, with hurricanes, wildfires, flooding, and compound climate shocks posing increasing threats to critical infrastructure. 1 The American College of Physicians has recommended that the healthcare community throughout the world engage in environmentally sustainable practices and support efforts to mitigate and adapt to the effects of climate change. 2 There are projections that the increased trajectory of climate change can lead to increased morbidity and mortality, along with decreased worker productivity. 3 These climate-driven disruptions can also rapidly compromise the medical supply network, creating dangerous shortages of vital resources, such as intravenous (IV) fluids, that healthcare clinicians depend on for patient treatment across various medical settings. In response to the increasing intensity of climate disasters, healthcare systems must strengthen supply chain resilience to safeguard uninterrupted access to essential medical supplies. 4

In response to mounting climate challenges, global and national health authorities are developing frameworks to strengthen healthcare system resilience. The World Health Organization has established a comprehensive operational framework with 10 key components for building climate-resilient health systems, emphasizing the dual responsibility to both prepare for climate impacts while reducing healthcare’s carbon footprint, which currently accounts for approximately 5% of global greenhouse gas emissions (GHG), and nearly 10% of all GHG emissions in the US. 5,6 Complementing these international efforts, the US Department of Health and Human Services (HHS) has developed its own climate resilience initiatives through the Administration for Strategic Preparedness and Response, including guidance for healthcare organizations on developing climate resilience plans that address infrastructure vulnerabilities, community partnerships, and protection of at-risk populations. 7

Central to both frameworks is the recognition that healthcare supply chains are integral components of resilient health systems. As part of its CARES (Climate Action, Resilience, and Equity Solutions) Pledge, HHS asked healthcare organizations to conduct an inventory of their supply chain emissions by the end of 2024 and collaborate with international partners on mitigation strategies. This reflects a broader shift toward a multidisciplinary approach to healthcare resilience—one that extends beyond emergency preparedness to include proactive climate risk assessment, supply chain diversification, and community-wide adaptation to maintain access to essential medical supplies during climate-related disruptions. These climate resilience frameworks have become increasingly urgent as real-world events demonstrate the vulnerability of healthcare supply chains to extreme weather.

Two recent disasters—Hurricane Maria in September 2017 and Hurricane Helene in September 2024, both Category 4 storms—led to critical nationwide shortages of IV fluids, exemplifying the risks of a highly centralized medical supply chain. When Hurricane Maria struck Puerto Rico, it caused severe damage to Baxter International’s manufacturing facilities, which supplied a significant share of the small-volume saline bags used in the US. This disruption led to widespread shortages across US hospitals, prompting the adoption of emergency conservation measures such as administering IV push antibiotics and oral rehydration strategies. 8,9 In 2024, Hurricane Helene severely damaged Baxter International’s North Cove facility in North Carolina—the largest manufacturer of IV fluids in the US, which produces about 60% of IV fluids used by US hospitals, 10 leading to an immediate nationwide shortage. 11 In response, the US Centers for Disease Control and Prevention issued a Health Alert Network advisory to encourage IV fluid conservation. 12 The American Society of Health-System Pharmacists published clinical guidance to help hospitals manage IV fluid shortages. 13

**Population Health Research Capsule**

What do we already know about this issue?

*Intravenous (IV) fluid shortages disrupt emergency care. Conservation strategies can preserve supply and reduce waste during national crises*.

What was the research question?

*Did large-volume IV fluid use decrease during the national shortage following implementation of communication strategies to reduce unnecessary IV fluid-bolus orders?*

What was the major finding of the study?

*Large-volume IV fluid-bolus orders fell from 41.8% of ED visits before, to 30.8% during the IV fluid shortage, potentially saving $109,000 per year 3.1 metric tons of CO_2_ emissions*.

How does this improve population health?

*System-level conservation reduces resource use and emissions, improving health system sustainability and preparedness for future shortages*.

**Figure 1:**
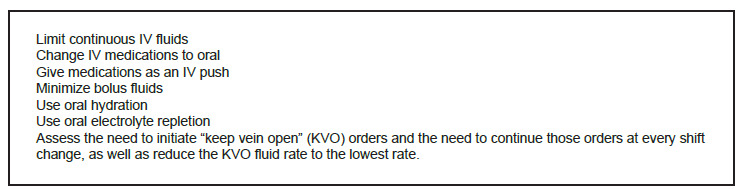
Mitigation strategies to limit intravenous fluid conservation when clinically appropriate during the national IV fluid shortage in October 2024. *IV*, intravenous.

Hospitals around the country quickly pivoted to create reduction protocols. Through the creation of an incident command structure, tiered communication huddles, and service line clinical practice guidelines, Intermountain Health achieved a 32% reduction across 34 sites within five weeks. 14 University hospitals in Cleveland cut IV fluid use by 65%, surpassing a 40% reduction target. 15 Our institution published a critical shortage alert, highlighting the need to reduce IV fluid use by 50% and provided several mitigation strategies (Figure 1). In this paper we focus on the ED and one mitigation strategy, minimizing bolus fluids, a common intervention in the ED. Conservation efforts focused on clinical decision-making and electronic health record (EHR) behavior. An interruptive alert was launched in our EHR (Epic Systems Corporation, Verona, WI) on October 17, 2024, requiring acknowledgment before proceeding with IV fluid-bolus orders. There were also frequent reminders during morning huddles to avoid unnecessary IV fluid use and consider oral rehydration when patients are able to tolerate it.

## METHODS

We conducted a retrospective analysis of large-volume IV bolus orders placed for adult patients (> 18 years) in three EDs between July 19, 2024–April 14, 2025. We included all patient encounters with large-volume (500 mL or 1,000 mL) bolus orders of either normal saline or lactated Ringer solution. Clinical orders placed during the ED visit were extracted from the Epic Clarity database. Distinct encounters were identified by contact serial numbers. We used descriptive statistics to compare the volume of IV fluids, associated costs, and estimated emissions across three time periods. Changes in proportions were expressed as relative percentage change, weighted by site-level patient encounter volumes.

The study period was divided into three time frames:

- Pre-shortage: July 19–October 16, 2024
- Critical shortage: October 17, 2024–January 14, 2025 (defined by initiation of the interruptive alert)
- Post-shortage: January 15–April 14, 2025 (defined by the deactivation of the interruptive alert)

We conducted a chi-square test of independence to assess whether the proportion of ED encounters involving large-volume IV fluid orders differed significantly across the study periods. The primary outcome was a binary measure: the presence or absence of a large-volume IV fluid order, defined as 500 mL or 1,000 mL of normal saline or lactated Ringer solution. All statistical analyses were performed using GraphPad Prism v10.5.0 (GraphPad Software, San Diego, CA), with statistical significance defined as *P* < .05.

**Figure 2:**
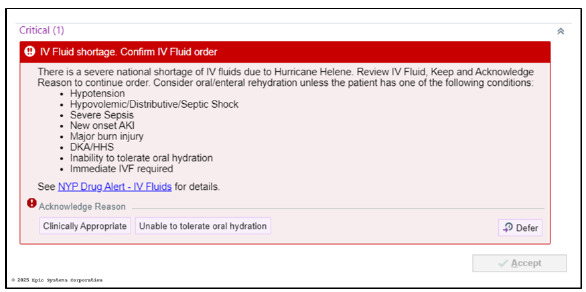
Interruptive alert in the electronic health record, prompting clinicians to justify intravenous fluid use based on a defined list of clinical indications.

The interruptive alert (Figure 2) required clinicians to confirm the necessity of IV fluids before ordering them and encouraged oral hydration when clinically appropriate. This intervention was paired with enterprise and department-wide communications, including email updates and clinical huddles. We identified orders for an oral hydration order introduced during the shortage period. Volume administered was not recorded in the EHR.

Cost Analysis: We estimated unit costs using the wholesale acquisition costs (WAC) obtained through a search on Micromedex RED BOOK (Merative, Ann Arbor, MI). 16 The WAC was used as a proxy for estimated market cost because institutional negotiated prices are proprietary and could not be disclosed. The WAC for 0.9% sodium chloride was $4.22 per 500-mL bag and $4.92 per 1,000-mL bag. For lactated Ringer, the WAC was $4.61 per 500-mL bag and $5.16 per 1,000-mL bag. To simplify the cost analysis, we averaged the WAC values for sodium chloride and lactated Ringer solution by volume and rounded to the nearest whole dollar. A unit cost of $4 was used for 500-mL bags, and $5 for 1,000-mL bags.

Environmental impact: We used life-cycle estimates of greenhouse gas emissions to calculate environmental burden: 390 grams (g) carbon dioxide (CO_2_) per 500-mL bag and 580 g CO_2_ per 1,000-mL bag. These values were obtained using published life-cycle data. 17 No differences in emissions were assumed between lactated Ringer solution and normal saline.

We incorporated relevant methodological principles described by Worster and Bledsoe (2005), 18 including the use of clear inclusion and exclusion criteria, a consistent case definition for eligible encounters, and standardized data abstraction from the EHR. This study was reviewed and approved by the Columbia University Irving Medical Center Institutional Review Board (IRB# AAAV7300) and was conducted in accordance with ethical standards for human subjects research. All data were de-identified before analysis.

## RESULTS

**Figure 3:**
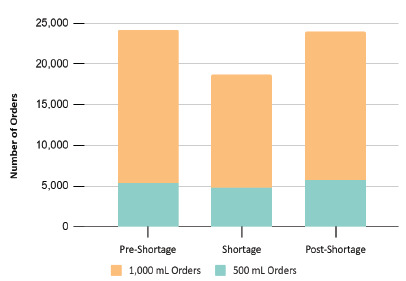
Total intravenous fluid orders by period and volume, reflecting changes in emergency physicians’ ordering behavior in response to shortages.

During the pre-shortage period, 24,251 IV fluid-bolus orders were placed across the three EDs. Orders dropped to 18,692 during the critical shortage, reflecting a 22.9% reduction. In the post-shortage period, orders rebounded to 23,911 but remained slightly below baseline (Figure 3). The reduction in IV fluid use was more pronounced among 1,000-mL bolus orders (−26.3%) compared with 500-mL bolus orders (−11.0%) during the shortage period, indicating that conservation efforts primarily affected larger volume infusions.

Large-volume IV fluids were ordered in 41.8% of ED encounters during the pre-shortage period, declining to 30.8% during the shortage period, and increasing to 39.6% in the post-shortage period (Table 1). The variation in ordering rates across these periods was statistically significant (χ^2^ = 1,181.0, *P* < .001). This relative reduction of 26.3% indicates a marked change in clinician behavior during the shortage. Following the resolution of the IV fluid shortage, this proportion rose to 39.7%, partially rebounding toward baseline. Compared to the pre-shortage period, the post-shortage proportion reflects a sustained relative reduction of 5.5%. These findings suggest a substantial and immediate shift in clinician behavior during the crisis, with evidence of some changes persisting after the acute intervention period.

Use of the oral hydration order was limited: 0 orders in the pre-shortage period; 30 during the shortage; and 23 in the post-shortage period. The total volume administered was not captured.

**Figure 4:**
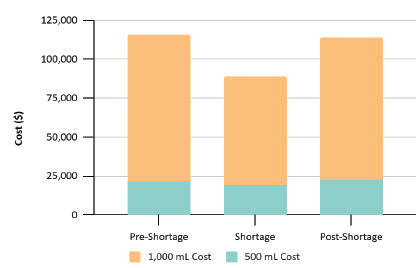
Total cost of intravenous fluids by period and volume across the study period.

Cost dropped from $115,873 in the pre-shortage period to $88,671 during the shortage, reflecting decreased IV fluid use and yielding a short-term savings of $27,202 (Figure 4). If sustained, this reduction would project to approximately $108,808 in annual cost savings.

**Figure 5:**
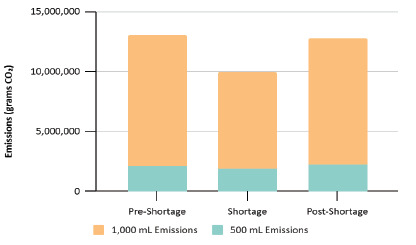
Carbon dioxide emissions from intravenous fluid use by period and volume.

Environmental impact decreased correspondingly. Intravenous fluid-related CO_2_ emissions fell from 13 metric tons in the pre-shortage period to 9.9 metric tons during the critical shortage, a 3.1 metric ton reduction (Figure 5). This is equivalent to eliminating the emissions of more than 349 gallons of gasoline. 19

## DISCUSSION

Our goal in this study was to assess the impact of an EHR-based interruptive alert and related interventions on IV fluid use, cost, and environmental emissions during and after a national shortage triggered by Hurricane Helene. Our findings also explore how these conservation strategies might support long-term improvements in healthcare sustainability and climate resilience. This study demonstrates that targeted conservation interventions are associated with rapid and substantial reductions in IV fluid use. The 22.9% drop in large-volume IV bolus orders observed during the critical shortage period, along with a sustained 5.5% relative reduction post-shortage, suggests that even short-term behavioral nudges, validated here through statistically significant changes, can have a durable impact on clinical practice.

Conservation strategies during a critical shortage may offer guidance for long-term change. Typically, conservation strategies prioritize critical care environments, including the ED. Our findings show that communication prompting clinicians to evaluate the clinical necessity of IV fluids, was associated with a meaningful reduction in use, even in emergency settings; however, the clinical impact of these reductions remains unknown. Reflexive reliance on IV hydration remains widespread and can contribute to fluid overload, electrolyte imbalances, unnecessary resource utilization, prolonged length of stay, and increased nursing workload—without improving patient outcomes. 20–22 Reducing inappropriate use can improve both patient safety and departmental efficiency.

Recent IV fluid shortages have exposed the fragility of centralized medical supply chains, particularly when production is concentrated in a limited number of domestic or offshore sites. Improving resilience requires strategies such as diversifying suppliers, expanding production capacity, and strengthening public-private coordination. 23 Our findings build on this effort by highlighting the importance of in-hospital conservation strategies, which can support broader efforts to prepare for and manage future supply disruptions.

## LIMITATIONS

Our cost analysis uses the WAC, which is set by the manufacturer and generally exceeds the actual cost hospitals pay after contract negotiations. However, this approach does not reflect the full cost of IV fluid use, which extends beyond product pricing. A national review of hospital pricing data found that a single 1,000-mL bag of normal saline could carry negotiated insurance prices around $70, and uninsured cash rates near $114. 24 Adding this perspective makes the potential impact of reduced utilization even more economically relevant, particularly for health systems and patients navigating high-cost care environments. Moreover, although outside the scope of this analysis, IV fluid administration also affects nursing workload, requires additional supplies, and may impact length of stay.

Our statistical approach was partially limited to descriptive analyses, including relative percentage changes across predefined time periods. The absence of a control group limits our ability to isolate the effects of the interruptive alert and associated interventions from other concurrent system-wide changes. We cannot exclude the possibility that clinicians had already begun conserving IV fluids before the interruptive alert. Additionally, our reliance on EHR data may under-represent fluid orders that were discontinued or not administered. Moreover, we did not adjust for potential confounders, including seasonal variation or patient acuity, all of which could influence ordering patterns.

This analysis did not evaluate associations between IV fluid use and patient outcomes, as defining, extracting, and interpreting such data retrospectively was beyond the scope of this study. The focus instead was on system-level resource metrics. As a result, we could not determine whether the reduction in IV fluid use affected clinical outcomes, either positively or negatively. Notably, there was no intervention requiring IV fluid administration to deviate from standard clinical indications, and all treatment decisions were made at the discretion of the treating clinician.[Table t2-wjem-27-250]

While an oral hydration order was introduced during the shortage it was used infrequently, and volume administered was not recorded. Oral rehydration was encouraged in the interruptive alert, but it did not direct the clinician to place an oral rehydration order. We were, therefore, unable to evaluate whether oral rehydration served as a meaningful clinical substitute for IV fluids.

## CONCLUSION

Conservation strategies implemented during a crisis can drive measurable and sustained reductions in IV fluid use. Future work should explore how such approaches can be extended beyond acute periods and applied to other resource-intensive areas of hospital operations. Further studies should assess the impact of IV fluid conservation on patient outcomes, including need for admission, length of stay, return visits, symptom improvement, and adverse events such as pulmonary edema and electrolyte abnormalities.

## Figures and Tables

**Table 1:** Proportion of emergency department encounters involving large-volume intravenous fluid orders by study period.

Time period	Total encounters	Encounters with fluids	Encounters without fluids	Proportion with fluids
Pre-shortage	41,752	17,466	24,286	41.8%
Shortage (interruptive alert active)	39,840	12,255	27,585	30.8%
Post-shortage	40,967	16,207	24,760	39.6%

**Table 2 t2-wjem-27-250:** Intravenous fluid usage, cost, and environmental impact by study period.

Pre-shortage period bolus volume (mL)	Number ordered	Volume cost ($)	Total cost ($)	Grams CO_2_ emissions per order	Total grams CO_2_ emissions	Total fluid volume given (L)
Pre-shortage period:						
500	5,382	4	21,528	390	2,098,980	2,691
1,000	18,869	5	94,345	580	10,944,020	18,869
Combined (1,000 and 500)	24,251		115,873		13,043,000	21,560
Shortage period:						
500	4,789	4	19,156	390	1,867,710	2,394
1,000	13,903	5	69,515	580	8,063,740	13,903
Combined (1,000 and 500)	18,692		88,671		9,931,450	16,297
Post-shortage period:						
500	5,677	4	22,708	390	2,214,030	2,838.5
1,000	18,234	5	91,170	580	10,575,720	18,234
Combined (1,000 and 500)	23,911		113,878		12,789,750	21,072

*CO_2_*, carbon dioxide.

## References

[1] National Oceanic and Atmospheric Administration (NOAA), National Centers for Environmental Information (2025). 2024: an active year of U.S. billion-dollar weather and climate disasters.

[2] Crowley RA (2016). Climate change and health: a position paper of the American College of Physicians. Ann Intern Med.

[3] Haines A, Ebi K (2019). The imperative for climate action to protect health. N Engl J Med.

[4] Lee H, Romero J, Core Writing Team (2023). Climate Change 2023: Synthesis Report. Contribution of Working Groups I, II and III to the Sixth Assessment Report of the Intergovernmental Panel on Climate Change.

[5] World Health Organization (2023). Operational Framework for Building Climate Resilient and Low Carbon Health Systems.

[6] World Health Organization (2023). WHO unveils framework for climate resilient and low carbon health systems.

[7] US Department of Health and Human Services (2023). Developing a Climate Resilience Plan for Healthcare Organizations: Key Considerations.

[8] Yagnik KJ, Brown LS, Saad HA (2022). Implementation of IV push antibiotics for outpatients during a national fluid shortage following Hurricane Maria. Open Forum Infect Dis.

[9] Patiño AM, Marsh RH, Nilles EJ (2018). Facing the shortage of IV fluids—a hospital-based oral rehydration strategy. N Engl J Med.

[10] Garcia A (2024). IV shortage update: Baxter facility damage after hurricane in North Carolina 2024.

[11] US Centers for Disease Control and Prevention (2024). Disruptions in availability of peritoneal dialysis and intravenous solutions from Baxter International facility.

[12] US Centers for Disease Control and Prevention (2024). Health Alert Network Advisory: IV fluid shortage.

[13] American Society of Health-System Pharmacists (2024). Small- and Large-Volume Fluid Shortages: Suggestions for Management and Conservation.

[14] Blagev DP, Shakespeare W, Grissom CK (2025). Intermountain Health’s IV fluid reduction in response to Hurricane Helene.

[15] Becker’s Hospital Review (2024). University hospitals achieved a 65% IV fluid reduction during national shortage.

[16] Corporation IBM (2025). IBM Micromedex RED BOOK [Internet database].

[17] Eckelman MJ, Huang K, Lagasse R (2020). Health care pollution and public health damage in the United States: an update. Health Aff (Millwood).

[18] Worster A, Bledsoe RD, Cleve P (2005). Reassessing the methods of medical record review studies in emergency medicine research. Ann Emerg Med.

[19] United States Environmental Protection Agency (2024). Greenhouse Gas Equivalencies Calculator.

[20] Cerceo EA, Deitelzweig SB, Sherman BM (2024). The impact of climate change–induced natural disasters on healthcare: rethinking intravenous fluids. J Hosp Med.

[21] National Institute for Health and Care Excellence Intravenous Fluid Therapy in Adults in Hospital (2023). NICE guideline [CG174].

[22] O’Connor ME, Prowle JR, Kirwan CJ (2016). Fluid overload is associated with increased mortality in critically ill patients. Nat Rev Nephrol.

[23] Davis AP (2025). Enhancing resilience in the healthcare supply chain: lessons from IV fluid shortages in the wake of natural disasters.

[24] Goodbill (2022). Is a hospital really charging $26,667 for a bag of saltwater?.

